# Meta-analysis of Psychotherapy for Autistic Youth

**DOI:** 10.1007/s10578-024-01686-2

**Published:** 2024-04-05

**Authors:** Kashia A. Rosenau, Junok Kim, An-Chuen Billy Cho, Michael Seltzer, Ana M. Ugueto, John R. Weisz, Jeffrey J. Wood

**Affiliations:** 1 David Geffen School of Medicine, University of California Los Angeles, Los Angeles, USA; 2 School of Education and Information Studies, University of California Los Angeles, Los Angeles, USA; 3 University of Texas Health Science Center at Houston, Houston, USA; 4 Harvard University, Cambridge, USA

**Keywords:** Autism, Psychotherapy, Meta-analysis, Cognitive behavioral therapy, Behavioral therapy

## Abstract

In order to provide more individualized support, it is imperative to further understand the effectiveness of different types of psychotherapy on the clinical areas of need common in autistic youth (Wood et al. in Behav Ther 46:83–95, 2015). Randomized controlled trials of psychotherapy for autistic youth were included if published in English, included random assignment to treatment or control group, required a previous diagnosis of autism, had a mean age of 6–17 years, and provided outcome measure data from both intervention and control groups. A total of 133 measures were coded across 29 studies and included 1464 participants with a mean age of 10.39 years (1.89). A small mean effect size (0.38,95% CI [0.26, 0.47]) was found overall, with the largest effects for cognitive behavioral therapies on autism-related clinical needs (0.81) and overall mental health (0.78). The results show the significant impact of psychotherapy interventions for autistic youth. Additional research should further assess the details of the most effective psychotherapies for each area of clinical need.

Many psychotherapy interventions and supports exist for autistic youth; however, little is known about the comparative effectiveness amongst the different types of psychotherapy. The range of outcome measures utilized in intervention studies for autistic youth is also limited, as researchers have overwhelmingly focused on internalizing problems, even though psychotherapy has shown to be helpful in numerous areas of clinical need [1, 2]. The purpose of the present study is to examine the impact of different types of psychotherapy on documented areas of clinical need for autistic youth. The results from this meta-analysis can be used to make informed decisions about which types of psychotherapy to implement for specific clinical needs for autistic youth.

## Autism

Autism is characterized by differences in communication and social interaction, as well as repetitive behaviors and actions and intense interests [3]. The current prevalence rate of autism in the United States is 1 in 36 children [4]. Autism is quite heterogenous as there is significant variability in the daily support needs of autistic individuals [5]. Many autistic individuals live very independent lives without support, while some autistic individuals have a few areas of support needs, and others require daily in-home support to meet their needs. The current *Diagnostic and Statistical Manual of Mental Disorders* (5th ed.; *DSM–5*) classifies autism on a spectrum [3].

### Areas of Clinical Need for Autistic Youth

Although three main characteristics exemplify autism (differences in communication, social skills, and inflexible behavior [3]), there are multiple areas of clinical need that can be addressed in psychotherapy. Previous research has identified six main areas of potential clinical need for autistic youth: externalizing problems, internalizing problems, repetitive behavior, peer social engagement, social communication, and self-care [1, 2].

Each of the six clinical areas of need may benefit most from distinctively different forms of psychotherapy. Autistic youth with internalizing problems, such as anxiety and depression, seem to benefit from systematic desensitization [6], while autistic youth with externalizing problems, such as aggression, seem to benefit from self-management interventions [7]. Cognitive behavioral therapy (CBT) has been shown to have a positive impact on both [1, 2, 8]. Knowing which types of psychotherapy are most effective for each area of clinical need is a necessary next step in creating more individualized treatment plans for autistic youth. A similar model has been applied in school settings identifying the evidence-based practices with research linked to specific outcomes for autistic youth [9]. If researchers know which areas of clinical need different types of psychotherapy can effectively target, then that information can be used to guide treatment selection for each individual based on their unique needs.

### History of Therapy for Autistic Youth

The lack of understanding of autism originally led many people to believe that it could or should be cured and many unsuccessful interventions were utilized for autistic youth [10]. Regardless of the presence of a co-occurring intellectual disability, many autistic youth were previously sent to schools for individuals who need substantial support due to intellectual disabilities, while other autistic youth were institutionalized. Only the truly wealthy received services for autism and, even then, it was focused more on management, such as reducing outbursts or repetitive behaviors, than on individual goals or positive development. Some interventions lacked evidence and others were actually damaging to autistic youth [10].

Issues with effective interventions were originally matched with issues in diagnosis. Autism can be difficult to accurately diagnose, as some individuals initially seem to show typical development and others are not detected until late childhood [11]. Improvements in diagnosis and diagnostic tools have occurred in the last decade and early diagnosis can now occur at 12 months of age [12]; however, advancements in diagnosis must be met with advancements in evidence-based interventions and supports.

Therapy and support for autistic individuals have moved away from the institutional approach and towards the unique needs of each individual. For example, CBT and behaviorally informed therapies (BITs), such as social skills training, are commonly used interventions for autistic youth. CBT has been shown to effectively reduce anxiety and depression, maladaptive behaviors, and improve self-care skills [13–17] and social skills training is effective in improving social competence, increasing social get-togethers, and reducing internalizing symptoms [18–20].

The negative history of therapy for autism highlights the need to ensure that efficacious interventions and supports are utilized and expanded moving forward. The increase in the neurodiversity movement, celebrating the natural diversity in neurotypes, has fostered an increase in autistic advocacy [21] and many autistic adults have voiced their concerns regarding certain forms of behavioral therapy [22]. Although CBT and behavioral therapies have many positive aspects, they must be consistently implemented in a respectful manner that focuses on the unique goals of each individual.

### Meta-analyses of Psychotherapy for Autistic Youth

Meta-analyses have been effective in summarizing decades of intervention research into a quantifiable measure of impact that can be assessed across studies in the past, present, and future. Most notably, Weisz et al. [23] analyzed fifty years of research on psychotherapy for children and adolescents. The analyses involved 447 studies that were synthesized to discover psychotherapy has a medium effect (0.46) for youth and those who received interventions had a 63% probability of improved outcomes over the comparison group. The results of the meta-analysis by Weisz et al. [23] raise important concerns in the field of youth psychotherapy that can help guide future intervention creation and modality. Additionally, the Weisz study created a robust model for meta-analytic work in this field, including an extensive codebook for meta-analyses on psychotherapy, which was utilized in the present study.

Previous meta-analyses on interventions and supports for autistic youth have typically focused on specific types of psychotherapy. For example, Weston et al. [24] performed a meta-analysis on cognitive behavioral therapy for autistic youth, while Gates et al. [18] performed a meta-analysis on group social skills interventions for autistic youth. A recent meta-analysis examined therapies across broader domains for early intervention studies [25]. The existing research in this area does not provide a full picture of the array of types of psychotherapy interventions and supports available, nor does it provide an evaluative comparison of the impact of different types of psychotherapy. In order to fill the gap in existing research, meta-analytic studies on psychotherapy for autistic youth must be expanded to include the evaluation of the impact of each type of psychotherapy on the areas of clinical need commonly identified in autistic youth.

### Purpose Statement

The purpose of the present study is to examine the evidence-base of interventions for autistic youth using a quantitative analytic approach that will synthesize the findings and ease comparison across studies. Effect sizes were computed for each of the areas of clinical need (externalizing problems, internalizing problems, repetitive behavior, peer social engagement, social communication, and self-care; [1, 2]) and types of psychotherapy (e.g. behaviorally-informed therapy and cognitive behavioral therapy). This is crucial, as the results are a quantifiable measure that can be compared across studies to examine the impact of different types of psychotherapy on each of the areas of clinical need. The results from this meta-analysis can be used to make informed decisions about interventions and supports to implement for specific areas of clinical need in autistic youth.

### Research Questions

1. What is the overall effect of psychotherapy for autistic youth?
  - The results can be compared to the robust meta-analysis of youth psychotherapy by Weisz and colleagues [23] to see if psychotherapy tends to be overall more or less effective for autistic youth.
2. Does therapy impact differ by type of psychotherapy?
  - Previous meta-analyses have included only CBT or social skills training; however, the present study will evaluate the impact across different interventions to see which types of psychotherapy are most effective and make a larger difference in the lives of autistic youth.
3. Does therapy impact differ by the areas of clinical need for autistic youth?
  - Not only can more personalized goals be targeted by knowing the clinical areas of need that are significantly impacted by psychotherapy for autistic youth, but the field of autism research benefits from also knowing which areas of clinical need are currently lacking sufficient support and warrant further scientific inquiry [1, 2].
4. Does the impact of each type of psychotherapy differ for each of the areas of clinical need in autistic youth?
  - Examining both the types of psychotherapy and clinical areas of need can assist in more precise treatment plans based on the needs of the individual and available interventions that effectively address those needs [1, 2]. Applying this model to the field of psychotherapy for autistic youth can potentially improve outcomes from therapeutic interventions.

## Methods

### Search Strategy and Study Selection

The present study evaluated randomized controlled trials (RCTs) of psychotherapy for autistic youth. RCTs were chosen to identify the best-case scenario for a research base supporting the use of psychotherapy for autistic youth. Psychotherapy treatment, as defined by Weisz et al. [23], includes “an approved form of psychotherapy or psychosocial treatment (i.e., intervention designed to alleviate non-normative psychological distress, or reduce maladaptive behavior, or increase deficient adaptive behavior through counseling, interaction, a training program, or a predetermined treatment plan).”

Three search engines and databases were utilized, PubMed, PsychInfo, and ERIC. The reference sections of relevant studies were also searched for additional studies appropriate for the meta-analysis. The following keywords were searched in all combinations “psychotherapy”, “intervention”, “therapy”, “youth”, “child”, “adolescent”, “autis*”, “Asperger”, “autism spectrum disorder” and “ASD”. Articles were prescreened by trained research assistants and screened by the primary author.

### Inclusion and Exclusion Criteria

Inclusion criteria include: (a) random assignment to a treatment or comparison group, (b) a previous diagnosis or confirmed diagnosis of autism or Asperger syndrome, (c) mean age of 6 to 18 years, (d) outcomes focused on autistic youth, (e) psychotherapy as the intervention, and (f) outcome measure data collected from both the intervention and control groups. The date of publication was not limited.

Publications covering single case studies were excluded, as well as those with main outcome measures not focused on autistic youth (e.g. outcome measures focused on caregivers), and those published as dissertations. Only studies published in English were included. Additionally, pharmacological studies were excluded. Studies involving social skills group training were excluded due to the substantial body of literature on programs in that field, for example the UCLA Program for the Education and Enrichment of the Relational Skills (PEERS; [20]), and numerous recent meta-analyses that cover social skills group training (e.g. [18, 26]).

### Study Coding

An extensive codebook from meta-analytic work by Weisz et al. [23] was adapted for the present study. Updates to the codebook included the addition of autism specific target problem codes that encompass autism, social communication differences, restrictive and repetitive behaviors, anxiety and autism, and emotion regulation and autism. The clinical areas of need were also added to the codebook [1, 2] along with autism-related clinical needs (e.g. measures such as the Social Responsiveness Scale) and general mental health outcomes (e.g. measures such as the Strengths and Difficulties Questionnaire)—due to the emergence of these areas during measure coding. Studies were coded for a subset of the variables outlined by Weisz et al. [23] and the additional autism specific target problem codes and clinical areas. Studies were coded using three separate coding sheet templates for variables related to the study, treatment and comparison groups, and outcome measures assessed.

#### Study Code Sheets

Variables included on the study code sheet pertain to the study methodology and participant demographics, such as the year of publication, mean age, gender, ethnicity, type of target problem, confirmation of diagnosis, single or multiple problems, and IQ score cutoff.

#### Group Code Sheets

A separate group code sheet was completed for each group (typically treatment and comparison) and include a description of the group, treatment code that encompasses the type of treatment or comparison group, treatment format, format of the session—individual or group, treatment integrity (pre-therapy training and adherence checks), and utilization of a treatment manual or protocol.

#### Measure Code Sheets

Each outcome measure with a post-treatment score for both the treatment and comparison groups was coded based on the name of the measure, clinical areas the measure covers, type of assessment, source of the rating, subject of rating, blindless of subject to the assessment, type of scores produced, scoring direction, and information related to the effect size statistic, such as the sample size, means, mean change scores, standard deviations and standard errors, when applicable.

### Data Analysis

#### Effect Size Calculation

Given that all the measures are reported in the continuous scale, the effect size of each measure, *d* [27], was computed by dividing the mean difference between treatment and control group by the pooled standard deviation, ${S}_{within}$. The pooled standard deviation was calculated based on the sample size of each group, ${n}_{1}$ and ${n}_{2}$, and the standard deviation of each group, ${{S}_{1}}^{2}$ and ${{S}_{2}}^{2}$. The error variance attached to each effect size, ${V}_{d}$, is obtained from ${n}_{1}$ and ${n}_{2}$, and *d* [28].

$$d{ } = { }\frac{{\overline{X}_{1} - \overline{X}_{2} }}{{S_{within} }},$$

where

$${S}_{within}=\sqrt{\frac{({n}_{1}-1){{S}_{1}}^{2}-({n}_{2}-1){{S}_{2}}^{2}}{{n}_{1}+{n}_{2}-2}}$$

$${V}_{d}=\frac{{n}_{1}+{n}_{2}}{{n}_{1}{n}_{2}}+\frac{{d}^{2}}{2({n}_{1}+{n}_{2})}$$

#### Multi-level Meta-analytic Approach

The analysis started with several univariate models and then proceeded to more complex models. Specifically, for each target clinical area, a 3-level multilevel model was specified to account for the variation of effect sizes across studies [29–31]. Mean effect sizes will be reported for clinical areas with three or more measures for a given type of psychotherapy. In total, there were eight analysis models to accommodate all clinical focus areas such as externalizing problems, internalizing problems, repetitive behavior, peer social engagement, social communication, self-care, autism-related clinical needs, and general mental health outcomes.

$${{\mathrm{Level}}-1: d}_{jk}={b}_{0jk}+{r}_{jk} , {r}_{jk} \sim N(0, {{s}_{{r}_{jk}}}^{2})$$

$${{\mathrm{Level}}-2: b}_{0jk}={\theta }_{00k}+{u}_{0jk} , {u}_{0jk }\sim N(0, {{s}_{u}}^{2})$$

$${{\mathrm{Level}}-3: \theta }_{00k}={\gamma }_{000}+{v}_{00k} , {v}_{00k}\sim N(0, {{s}_{v}}^{2})$$

$${d}_{jk}:\mathrm{ the\,observed\,jth\,effect\,size\,measure\,of\,study\,k}$$

$${b}_{0jk}:\mathrm{ the\,true\,value }(\mathrm{population\,value})\mathrm{ of\,jth\,effect\,size\,of\,study\,k}$$

$${r}_{jk}:\mathrm{ the\,random\,deviation\,for\,jth\,effect\,size\,of\,study\,k }(\mathrm{from\,the\,true\,value})$$

$${\theta }_{00k}:\mathrm{ the\,average\,of\,all\,effect\,sizes\,of\,study\,k}$$

$${u}_{0jk}:\mathrm{ the\,random\,deviation\,of\,jth\,effect\,size\,of\,study\,k }(\mathrm{from\,the\,average})$$

$${\gamma }_{000}:\mathrm{ the\,overall\,average\,across\,all\,studies}$$

$${v}_{00k}:\mathrm{ the\,random\,deviation\,of\,kth\,study }(\mathrm{from\,the\,overall\,average})$$

At level-1, the observed effect size of study k for outcome j, ${d}_{jk}$, is a function of true effect size or population value, ${b}_{0jk}$, and the random deviation, ${r}_{jk}$. The random effect ${r}_{jk}$ is assumed to follow a normal distribution with mean 0 and variance ${{s}_{{r}_{jk}}}^{2}$. Typically, ${{s}_{{r}_{jk}}}^{2}$ is the squared standard error reported with effect size measure ${d}_{jk}$.

At level-2, the true effect size of study k for outcome j,${b}_{0jk}$, is the sum of the average effect size for outcome j,${\theta }_{00k}$, and the random effect,${u}_{0jk}$. The random effect for outcome j is normally distributed with mean 0 and effect-size variance${{s}_{u}}^{2}$.

At level-3, the average of all effects sizes for study k, ${\theta }_{00k}$, is the sum of the overall average across all studies, ${\gamma }_{000}$, and the random deviation of the kth study, ${v}_{00k}.$

$${{\mathrm{Level}}-1: d}_{jk}={b}_{0jk}+{r}_{jk} , {r}_{jk} \sim N(0, {{s}_{{r}_{jk}}}^{2})$$

$${{\mathrm{Level}}-2: b}_{0jk}={\theta }_{00k}+{u}_{0jk} , {u}_{0jk }\sim N(0, {{s}_{u}}^{2})$$

$${{\mathrm{Level}}-3: \theta }_{00k}={\gamma }_{100}*(Psychotherapy1)+{\gamma }_{200}*(Psychotherapy)+ {\gamma }_{300}*(Psychotherapy3)+{v}_{00k} , {v}_{00k}\sim N(0, {{s}_{v}}^{2})$$

$${\gamma }_{100}-{\gamma }_{300}:\mathrm{ the average effect size of psychotherapy }1-3$$

At level-3 in the conditional model, above, the study-level indicator of the type of psychotherapy is included. The indicator, type of psychotherapy, is dummy-coded so that the coefficients, ${\gamma }_{100}-{\gamma }_{300}$, represent the mean effect size of each category across studies. For example, if the treatment of study k belongs to the first psychotherapy, then the average of effect ${\theta }_{00k}$ is the sum of ${\gamma }_{100}$, the average effect size of CBT studies, and the random deviation, ${v}_{00k}$, ${\theta }_{00k}={\gamma }_{100}*1+ {\gamma }_{200}*0+ {\gamma }_{300}*0+{v}_{00k}$.

Given low power to detect differences and the potential for unreliable findings, moderator tests were not conducted.

## Results

### Study Pool

**Fig. 1:**
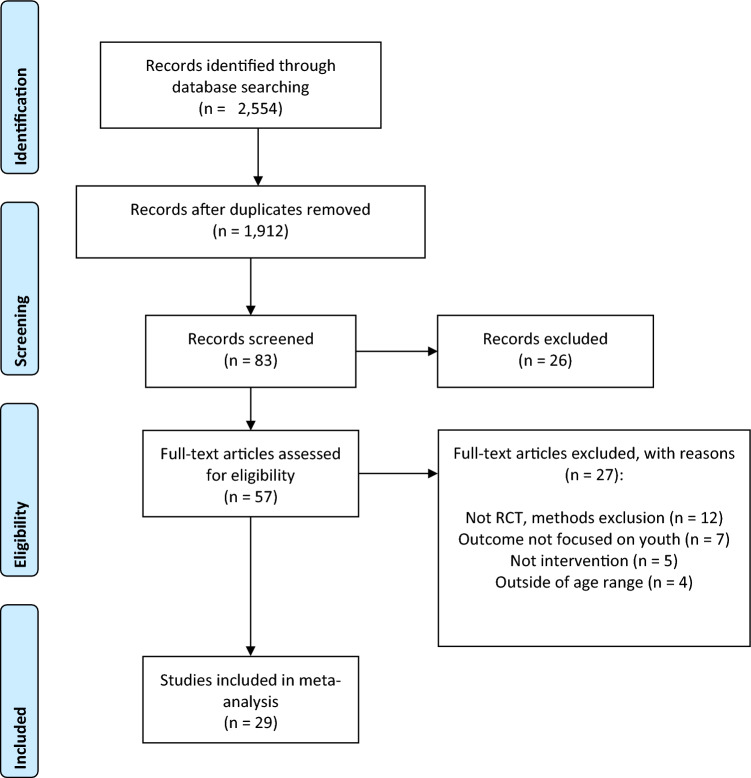
PRISMA flow diagram

Figure 1 presents the PRISMA flow diagram beginning with 2554 studies. After screening, a total of 133 measures were coded across 29 studies (listed in Table 1 and noted with an asterisk in the references) published between 2005 and 2021. One main coder independently coded all studies and 10% of studies were coded by another trained coder with an interrater agreement of 89%. The mean age of the 1464 participants is 10.39 years (1.89), with a minimum age of 7.86 and maximum of 13.39. The participants were overwhelmingly male (85%) and Caucasian (65%), although half of the studies did not report race/ethnicity. The average IQ was reported by 10 studies with a mean of 103.28 (9.17). CBT was the most common psychotherapy utilized (21 studies), then BIT (3 studies). The remaining studies were coded into an “other” category that consisted of distinctive interventions, such as therapeutic horseback riding, theatre interventions, and Lego therapy.

**Table 1:** Studies included in meta-analysis

First Author Last Name	Year	Age Range	Mean Age	Intervention Category
Andrews [46]	2013	7–12	9.02	CBT
Bass [47]	2009	4–10	7.34	Other
Chan [48]	2013	6–17	11.85	Other
Gabriels [49]	2015	6–16	10.2	Other
Gordon [50]	2015	9–14	11.45	Other
Kasari [35]	2012	6–11	8.14	BIT
Keehn [51]	2013	8–14	11.26	CBT
Koning [52]	2013	10–12	11.07	CBT
Maskey [53]	2019	8–14	10.79	CBT
Owens [54]	2008	6–11	8.18	Other
Reaven [55]	2012	7–14	10.44	CBT
Rice [34]	2015	5–11	7.77	BIT
Santomauro [56]	2016	13–18	15.75	CBT
Sofronoff [57]	2005	10–12	10.64	CBT
Sofronoff [8]	2007	10–14	10.78	CBT
Solomon [33]	2008	5–12	8.15	BIT
Soorya [58]	2015	8–11	9.96	CBT
Storch [59]	2013	7–11	8.89	CBT
Storch [60]	2015	11–16	12.74	CBT
Sung [61]	2011	9–16	11.21	CBT
Waugh [62]	2015	6–13	9	CBT
Weiss [63]	2018	8–12	9.75	CBT
White [64]	2013	12–17	14.58	CBT
Wijnhoven [65]	2020	8–16	11.1	CBT
Wood [66]	2009	7–11	9.2	CBT
Wood [40]	2014	7–11	8.8	CBT
Wood [1]	2015	11–15	12.3	CBT
Wood [17]	2020	7–13	9.9	CBT
Wood [67]	2021	6–13	9.56	CBT

The studies listed above are indicated with an * in the references section

### Posttreatment Results

The mean effect size across studies is 0.38; 95% CI [0.26, 0.47]. Table 2 presents the total estimated effect sizes for each of the clinical areas and types of psychotherapy, as well as the unique effect of each type of psychotherapy on each of the clinical areas of need with 3 or more measures per cell.

**Table 2:** The effect of different types of psychotherapy on clinical areas of need in autistic youth

	Clinical areas of need	Types of psychotherapy			
		Cognitive behavioral therapy	Behaviorally-informed therapy	Other	Total
Externalizing problems	Number of measures	5	6	2	13
	Number of studies	4	1	2	7
	Mean effect	0.32	–	–	0.59
Internalizing problems	Number of measures	51	1	3	55
	Number of studies	15	1	2	18
	Mean effect	0.45	–	–	0.43
Repetitive behavior	Number of measures	2	0	1	3
	Number of studies	2	0	1	3
	Mean Effect	–	–	–	− 0.02
Peer social engagement	Number of measures	14	8	7	29
	Number of studies	5	3	3	11
	Mean effect	0.15	0.05	0.25	0.15
Social communication	Number of measures	3	1	4	8
	Number of studies	3	1	3	7
	Mean effect	0.37	–	0.28	0.28
Self-care	Number of measures	1	0	1	2
	Number of studies	1	0	1	2
	Mean effect	–	–	–	–
Autism-related clinical needs	Number of measures	5	2	3	10
	Number of studies	5	1	2	8
	Mean effect	0.81	–	–	0.70
General mental health	Number of measures	4	1	4	9
	Number of studies	4	1	2	7
	mean effect	0.78	–	–	0.63
Total	Number of measures	85	19	25	133
	Number of studies	21	3	5	29
	Mean effect	0.42	0.49	0.24	0.38

#### Impact of Specific Types of Psychotherapy

A similarly small effect was found for both BITs (0.49; 95% CI [0.11, 0.73]) and CBT (0.42; 95% CI [0.26, 0.53]), while other interventions (0.25; 95% CI [0.07, 0.47]) also produced a small effect for autistic youth.

#### Impact on the Clinical Areas of Need

**Fig. 2:**
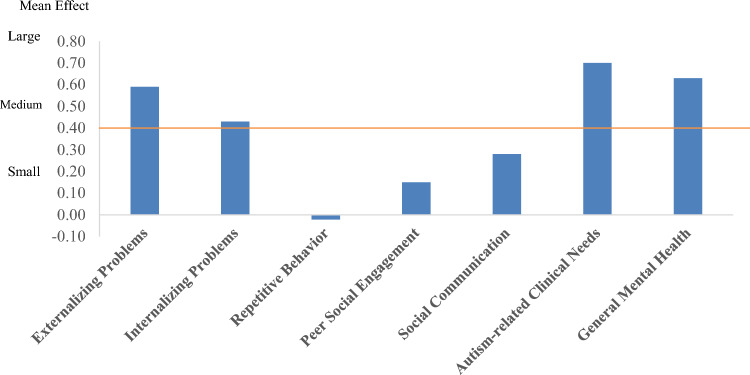
Mean effect sizes posttreatment by clinical areas of need. *Note* Dashed horizontal line shows the mean effect across all clinical areas

As shown in Fig. 2, the impact of psychotherapy is highest in autism-related clinical needs with a medium effect of 0.70 (95% CI [0.41, 0.92]). A medium effect was also found for general mental health (0.63; 95% CI [0.18, 1.11]) and externalizing problems (0.59; 95% CI [0.19, 0.74]), while small effects were found for internalizing problems (0.43; 95% CI [0.23, 0.50]), social communication (0.28; 95% CI [0.07, 0.45]), peer social engagement (0.15; 95% CI [0.00, 0.36]), and repetitive behaviors (− 0.02; 95% CI [− 0.036, 0.41]).

#### Impact of Specific Types of Psychotherapy on Clinical Areas of Need

CBT has a large impact on autism-related clinical needs (0.81; 95% CI [0.50, 1.23]) and a medium impact on general mental health outcomes (0.78; 95% CI [0.43, 1.13]). BITs have a small impact on peer social engagement (0.05; 95% CI [− 0.39, 0.38]). The estimated effect sizes for other interventions were in the small range for social communication (0.28; 95% CI [− 0.01, 0.47]) and peer social engagement (0.25; 95% CI [− 0.11, 0.62]).

## Discussion

What do we know about psychotherapy for autistic youth? While the present study provides insight into the impact CBT and BITs have on the areas of clinical need for autistic youth, it also highlights the need for both more precise evaluations of the types of psychotherapy and also the need for an increase in outcome measures related to adaptive skills and overall well-being. The comparable effect sizes between all youth in previous research and autistic youth in the present study suggests psychotherapy as a useful tool to increase the quality of life for all youth in need of clinical support.

### Impact of Specific Types of Psychotherapy

The overall small effect found for CBT with autistic youth in the present study is comparable to the small to medium effect found in previous meta-analytic work specifically focused on CBT [24]; however, a large effect was previously reported for CBT for autistic youth with anxiety [14]. The small effect size for BITs is in line with a recent meta-analysis which also found small effect sizes for behavioral therapies in autistic children [25]. The types of psychotherapy categories were created based on the interventions utilized in the coded studies; however, the majority of studies included in the meta-analysis were CBT interventions (21 out of 29) with only a few studies in the BIT (3) and other intervention groups (5).

The three BITs included in the analyses were focused on reinforcing positive behavior through parent interactions (Parent Child Interaction Therapy by [32, 33]), teaching face processing skills using a computer-based game [34], and didactic instruction and role playing [35]. These three therapies are distinctively more behaviorally focused and include fewer or no cognitive therapy elements compared to the CBT studies in the model. Additionally, these BITs are heterogenous and have relatively little in common. In order to more accurately evaluate the types of psychotherapy, it is important to dive deeper into the interventions included in each study, especially the elements that make up each intervention.

One valuable way to assess the impact of each intervention and facilitate comparison between modalities, is by categorizing psychotherapy based on the components utilized in each intervention. Odom [36] and Wong et al. [9] assessed the literature on evidence-based practices (EBP) utilized in interventions for autistic youth in single case, quasi-, and experimental designs, and the evidence of the efficacy of each EBP on outcome variable categories. Similarly, Chorpita et al. [37] outline the benefits of understanding the evidence-based practice elements used for youth psychotherapy.

Practice elements can be thought of as the ingredients that make up the intervention [37]. Research surrounding practice elements enables more personalized therapy plans based on individual goals, demographics, and social determinants. Examples of evidence-based practice elements are: directed play, modeling, psychoeducation for both families and youth, relaxation, cognitive coping, and exposures. Expanding the work by Chorpita and colleagues to evaluate practice elements utilized in psychotherapy for autistic youth can facilitate more precise evaluation of the magnitude of the impact of psychotherapy. On an individual level, this information can help guide more precise intervention selection based on an individual's unique needs. On a population level, adopting an evaluation of practice elements approach to psychotherapy research for autistic youth can help push the field to evaluate the allocation of resources towards areas in need of further scientific inquiry.

### Impact of Psychotherapy for Autistic Youth

The mean effect size of psychotherapy in autistic youth is 0.38. Weisz et al. [23] reported 0.46 from studies encompassing all youth, showing that psychotherapy is similarly as effective for autistic youth as it is for all youth. One of the major barriers to psychotherapy for autistic youth that was cited across coded studies is access to providers [38]. While many community therapists do not feel competent seeing autistic youth, those who do are often lacking quality resources for education and improvement in the delivery of services [38]. A shift in the practice of care to a more public health viewpoint—looking at disability from both a population health and individual needs perspective [39]—could increase the availability of providers for all youth. For example, the intervention manual for Schema, Emotion, and Behavior-Focused Therapy for Children (SEBASTIEN; 70) has been adapted to an online training platform that is freely available to practitioners wanting to increase their knowledge in CBT for autistic youth (Modular Evidenced Based Practices for Youth with Autism, MEYA, meya.ucla.edu).

### Impact on the Clinical Areas of Need

Two additional areas were added during the coding process to the original clinical areas outlined by Wood, McLeod, and colleagues [40]. Autism-related clinical needs and general mental health areas were added to include measures such as the Social Responsiveness Scale total score (SRS; [41]) and Strengths and Difficulties Questionnaire (SDQ; [42]); respectively. The SRS was one of the most commonly used measures across all studies and overall autism-related clinical needs shows the largest effect (0.70) across all of the clinical areas. There was an unfortunate lack of measures related to adaptive functioning in the literature base. An increase in the adoption of measures of self-care skills, peer friendship and inclusion, and overall well-being would provide helpful information on the impact of psychotherapy on the daily lives of autistic youth.

Moving forward, including a stronger neurodiversity lens to the field of psychotherapy for autistic youth, especially regarding language and outcome measurement, can help decrease stigma and increase collaboration between non-autistic researchers, autistic researchers, and the autistic community [43]. Valuable resources in community participatory autism research have been produced by the Academic Autism Spectrum Partnership in Research and Education (AASPIRE [44]) and infrastructure to ease data collection and promote interdisciplinary autism research on mental and physical health from a neurodiversity perspective is under development by the Autism Intervention Research Network on Physical Health (AIR-P [45]).

### Impact of Specific Types of Psychotherapy on Clinical Areas of Need

Table 2 presents the impact of each type of psychotherapy on each of the clinical areas of need. Autism-related clinical needs and general mental health have the highest effect sizes in the present study when CBT was received. The number of studies utilizing BITs and other interventions were small, thus the results must be interpreted with caution. The present results show CBT as effective in improving general mental health and decreasing autism-related clinical areas of need.

As outlined in the subsections above, important next steps in improving psychotherapy for autistic youth include using practice elements to evaluate interventions by the clinical areas of need they significantly impact and including more measures of adaptive skills focused on improving the quality of daily life for autistic youth. Improvements in these two areas would greatly increase the precision in which clinicians can measure the impact of specific methods on improving the well-being of autistic youth. This would allow for more personalized interventions to be developed based on each unique individual’s areas of clinical need and the interventions that include the practice elements most effective in improving those specific clinical areas.

#### Limitations and Future Directions

Codes for the different types of psychotherapy were based on the interventions described in the studies, which resulted in a CBT group, a small group of heterogenous BITs and a category of different interventions that did not fit together, nor did they fit in the CBT or BIT categories. The grouping of these categories does not produce especially meaningful results. Additional research is needed in many of the other intervention areas (theatre interventions, therapeutic horseback riding) before those studies can be meaningfully included. Additionally, while some of the clinical areas included an adequate—but still relatively low—number of coded measures, many did not; thus, full moderator analyses examining the types of psychotherapy and areas of clinical need were not feasible, and the current results are limited.

There is also a need to further evaluate the variance in each of the clinical areas to understand what is driving the mean effect sizes and if differences in impact are apparent based on the different measures used in a given clinical area or additional factors related to the specific studies or types of psychotherapy included in each clinical area of need. In order to assess the psychotherapies included in each clinical area, the studies must be examined on factors such as demographic characteristics, type of control group, therapist training, and duration of psychotherapy. This additional information would assist in the interpretation of the effect size in each clinical area of need and is an important next step.

## Summary

The results of the present study highlight the utility of psychotherapy to increase the well-being of autistic youth; as well as the need for more precision in evaluating different types of psychotherapy. As the growing body of research on psychotherapy for autistic youth continues, additional inquiry is warranted on the accuracy at which interventions can be personalized for the unique needs of each individual. Considering the comparable effect sizes of psychotherapy for all youth and specifically autistic youth, improving the precision of psychotherapy interventions could increase the quality of care for all youth in need of clinical support.

## Data Availability

The data that support the findings of this study are available from the corresponding author, KR, upon reasonable request.
